# Home Blood Pressure Monitoring in Primary Care: An Essential Approach to Hypertension Management

**DOI:** 10.7759/cureus.93163

**Published:** 2025-09-24

**Authors:** Eduardo D Martins, Alexandrina Martins, Catarina Rodrigues, Catarina Fernandes, Catarina Cascais, Gabriela Salvado dos Santos, Tatiana Raposo, Carolina Portela, Gonçalo NMO Silva, Bruno M Cerca, Filipe C Vasconcelos, Jaime Ribeiro, Nuno Amaral, Márcia C Sá, Tiago Mendes

**Affiliations:** 1 Family Medicine, USF Corgo, Unidade Local de Saúde de Trás-os-Montes e Alto Douro, Vila Real, PRT; 2 Family Medicine, USF Nova Mateus, Unidade Local de Saúde de Trás-os-Montes e Alto Douro, Vila Real, PRT; 3 Family Medicine, Unidade Local de Saúde de Trás-os-Montes e Alto Douro, Vila Real, PRT; 4 Family Medicine, USF Corgo, Vila Real, PRT

**Keywords:** blood pressure monitoring ambulatory, hypertension, patient engagement, primary health care, self care

## Abstract

Introduction

Home blood pressure monitoring (HBPM) has emerged as an essential tool for the diagnosis and management of arterial hypertension (AH). This study aimed to assess the role of HBPM as a complementary tool for the diagnosis of AH.

Methods

A retrospective study was conducted by analyzing a database of 189 patients from USF Corgo, who underwent HBPM between March and September 2023. After applying the exclusion criteria, the final sample consisted of 158 patients. A follow-up questionnaire was subsequently administered to 82 participants, addressing health literacy, therapeutic adherence, frequency of BP monitoring, and lifestyle modification measures.

Results

Of the 158 patients, 39 (24.7%) had controlled hypertension, 66 (41.8%) had uncontrolled hypertension, two (1.3%) had hypotension, 17 (10.8%) were newly diagnosed with AH, 32 (20.3%) exhibited white coat hypertension, and two (1.3%) did not have hypertension despite the presence of target organ damage. HBPM had a statistically significant impact on therapeutic adherence (p=0.007), changes in exercise habits (p=0.008), and the frequency of BP monitoring (p<0.001).

Conclusions

This study highlights the relevance of HBPM as a tool for detecting white coat hypertension and uncontrolled AH. Furthermore, HBPM was shown to significantly improve therapeutic adherence, BP self-monitoring, and the adoption of a healthier lifestyle.

## Introduction

Arterial hypertension (AH) is defined as a sustained elevation of systolic blood pressure (SBP) and/or diastolic blood pressure (DBP), with cut-off values varying according to the context where it is assessed (office or outpatient setting) [1]. AH is considered one of the main risk factors for the development of cardiovascular disease (CVD), which in turn is a leading cause of morbidity and mortality. Proper diagnosis, monitoring, and treatment are essential for effective risk control [2]. Office blood pressure (BP) measurement has numerous limitations: on the one hand, the limited number of measurements taken and, on the other, patient anxiety during medical or nursing appointments, which often leads to overestimation of BP values and inaccurate AH diagnosis [2]. To overcome these shortcomings, two diagnostic methods have been highlighted: home BP monitoring (HBPM) and ambulatory BP monitoring (ABPM). Although ABPM is considered the gold standard for diagnosing AH, its high cost and limited accessibility restrict its routine use [3].

HBPM involves the measurement of BP at home, by the patient or with the assistance of others. It involves taking consecutive BP readings by using a validated automatic sphygmomanometer at different times of the day and over several days. Its reproducibility has made it an attractive diagnostic method for managing this chronic disease, thus reducing the use of primary healthcare resources [4]. Furthermore, HBPM can detect the phenomenon of white coat hypertension, thereby avoiding the initiation of potentially harmful medications [5]. It is worth underscoring that iatrogenesis is the fifth leading cause of death worldwide [6]. Regarding cardiovascular events, HBPM - when compared with office BP measurement - shows a stronger association with target organ damage (TOD) and is a better predictor of such events and overall mortality [7].

Adherence to medical recommendations regarding lifestyle changes and pharmacological treatment is often low among patients [8]. In a 2004 study, adherence to antihypertensive therapy ranged from 50 to 70% [9]. One of the reasons for non-adherence may be related to the silent nature of AH, which typically lacks symptoms that would confirm improved BP control, potentially leading to lower compliance with prescribed measures [10]. Although the topic remains controversial, HBPM emerges as a potentially effective strategy to improve treatment adherence, as it enables patients to recognize elevated BP values at home rather than relying solely on office readings [10]. 

Most scientific studies on AH rely on office BP measurements, despite the known limitations recognized by various scientific societies [11,12]. In this context, literature on HBPM remains relatively scarce. Hence, there is a clear need to further investigate and generate robust scientific evidence, including the potential role of HBPM in enhancing treatment adherence. The primary objective of this study was to analyze the impact of HBPM as a complementary tool for diagnosis and therapeutic management. Additionally, this study aims to evaluate the impact of HBPM on health literacy, adherence to home BP monitoring, and adherence to antihypertensive medication.

## Materials and methods

Study design

This observational study was carried out at USF Corgo, a primary care family health unit (FHU) located in northern Portugal, which serves a mixed urban and rural population and is integrated into the public national health service. It aimed to analyze a database of patients who were prescribed and underwent HBPM, as part of an internal protocol developed at FHU, named “Control Hypertension at Home”, between March and September 2023.

Inclusion criteria: patients over 18 years of age, registered at the FHU, who were prescribed HBPM during the study period. Exclusion criteria: patients whose HBPM was deemed invalid according to validation procedures defined by the European Society of Hypertension (ESH) [11].

Ethical considerations

This study was conducted in accordance with the ethical principles of the Declaration of Helsinki 2013. The project was reviewed and approved by the Ethics Committee of the Unidade Local de Saúde Trás-os-Montes e Alto Douro (CES 5106). As the study involved retrospective data and noninvasive procedures, written informed consent was not required; however, verbal informed consent was obtained from all participants before the distribution of the follow-up questionnaire. All data were anonymized and processed in accordance with data protection regulations, ensuring strict confidentiality and privacy of patient information.

Data collection

**Figure 1:**
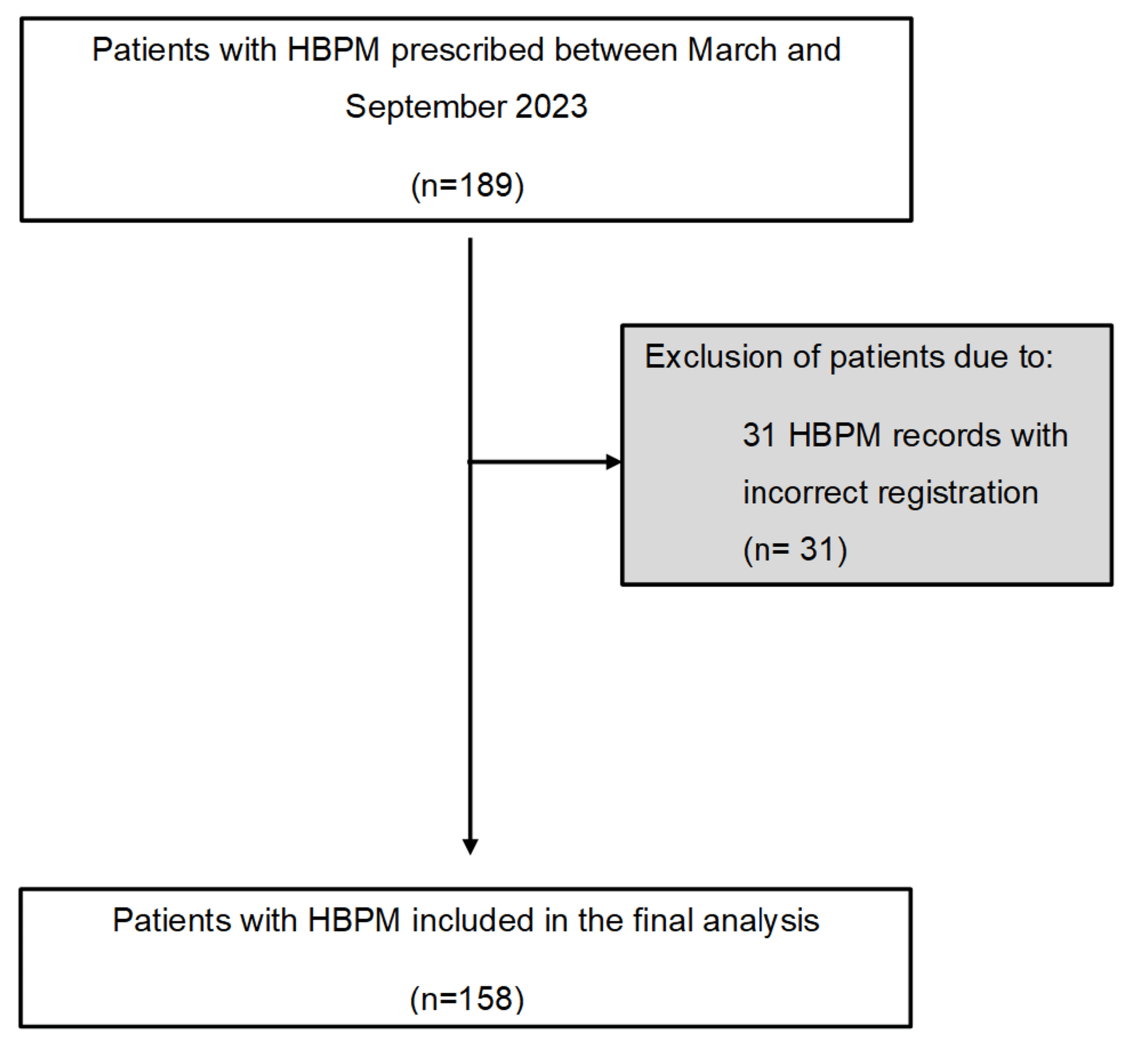
Flowchart depicting HBPM cohort selection HBPM: home blood pressure monitoring

Between March and September 2023, a total of 189 HBPMs were prescribed. Of these, 31 were excluded for not meeting the validation criteria (Figure 1). After selection, the following parameters were collected to characterize the population and clinical profile: age, sex, education level, BMI, smoking habits, and presence of other cardiovascular comorbidities such as diabetes mellitus (DM), dyslipidemia, and TOD (stroke, nephropathy, structural heart disease, atherosclerotic disease, retinopathy). For the diagnostic approach, the following data were collected: reason for HBPM prescription, mean SBP and DBP, and diagnostic conclusion based on HBPM.

To characterize the therapeutic approach, antihypertensive treatment-related data were collected before and after HBPM. To assess the impact of HBPM, a non-validated questionnaire was developed addressing health literacy related to hypertension, adherence to pharmacological therapy, frequency of HBPM, and lifestyle modification measures, such as salt intake and physical exercise (defined as at least 30 minutes of moderate-intensity aerobic exercise-walking, running, or cycling - five to seven days per week), before and after the use of the HBPM tool. The questionnaire was distributed by the research team in September 2024 via telephone contact, following verbal consent from each participant. The total number of participants was 82. Data were collected using the SClínico® software and information provided directly by patients. Data were entered into Microsoft Office Excel® 2021 spreadsheets.

HBPM Methodology

**Figure 2:**
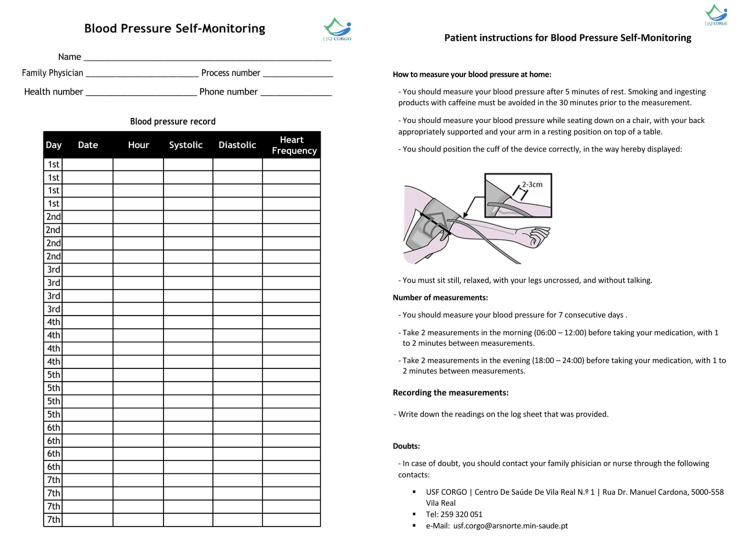
HBPM recording sheet and patient instructions HBPM: home blood pressure monitoring

Patients measured their BP at home using validated and calibrated automatic sphygmomanometers (according to STRIDE BP organization), provided by the FHU - model Omron M3 Comfort® (HEM-7134-E). Standard-size cuffs were used [1,11]. The validity of HBPMs was ensured in accordance with ESH recommendations [11]. During medical appointments, patients were instructed on the correct technique for HBPM. Measurements were recorded on a dedicated form provided at the time of prescription, which included instructions for correct BP measurement on the reverse side (Figure 2). After excluding the first day of measurements, average SBP and DBP values were calculated and recorded in Microsoft Excel® by the group of investigators.

Clinical justification for HBPM request

The sample was divided into three groups according to the reason for HBPM prescription. Follow-up group included patients with a prior diagnosis of AH in whom HBPM was requested based on clinical criteria (e.g., office BP above target, symptoms suggestive of low BP, need for treatment adjustment, new TOD, or other situations considered relevant by the prescribing physician). The diagnostic group included patients with elevated office BP values prompting HBPM. The previous TOD group included patients with existing TOD but no prior diagnosis of AH.

Diagnostic categorization

Definitions were based, when applicable, on ESH and European Society of Cardiology (ESC) recommendations for HBPM [1,11,12]. In cases where specific reference values were unavailable, the investigators applied thresholds similar to those recommended for office BP measurements by the ESC 2021 and ESC/ESH 2018 guidelines [1,12]. Controlled hypertension: previously diagnosed hypertensive patient with mean SBP <130 mmHg and DBP <80 mmHg; uncontrolled hypertension: previously diagnosed hypertensive patient with mean SBP ≥130 mmHg or DBP ≥80 mmHg; low BP: antihypertensive-treated patient with mean SBP <100 mmHg; newly diagnosed hypertension: HBPM with mean SBP ≥135 mmHg or DBP ≥85 mmHg; white coat hypertension: office BP ≥140/90 mmHg and HBPM showing mean SBP <135 mmHg and DBP <85 mmHg; normal BP: office BP <140/90 mmHg and HBPM with SBP <135 mmHg and DBP <85 mmHg; masked hypertension: office BP <140/90 mmHg with HBPM showing SBP ≥135 mmHg or DBP ≥85 mmHg [1,12].

Statistical analysis

Statistical analysis was performed using SPSS® Statistics version 25 (IBM Corp., Armonk, NY). A p-value <0.05 was considered statistically significant. Regarding descriptive statistics, categorical variables were presented as absolute (n) and relative (%) frequencies, and continuous variables as means and standard deviations (SD). As for inferential statistics, normality of quantitative variables was tested with the Kolmogorov-Smirnov (K-S) test. The Wilcoxon signed-rank test (Z) was used to assess changes in therapeutic adherence, BP monitoring frequency, and physical exercise before and after HBPM. The student’s t-test (t) was used to compare SBP and DBP across diagnostic and follow-up groups. Chi-square and ANOVA tests were used to analyze categorical and continuous variable distributions across groups, showing increased, maintained, or decreased habits regarding BP monitoring, medication adherence, and physical exercise.

## Results

Sociodemographic and clinical characteristics of the sample

The sociodemographic and clinical characteristics of the patients (n=158) included in the study are presented in Table 1.

**Table 1:** Sociodemographic and clinical characteristics of the sample SD: standard deviation; BMI: body mass index; HBPM: home blood pressure monitoring; SBP: systolic blood pressure; DBP: diastolic blood pressure

Sociodemographic and clinical characteristics	
Sex, n (%)	
Male	80 (50.6%)
Female	78 (49.4%)
Age, years, mean ± SD	61.5 ± 12.8
Education level, n (%)	
4th grade	52 (32.9%)
6th grade	29 (18.4%)
9th grade	25 (15.8%)
12th grade	31 (19.6%)
University education	21 (13.3%)
BMI, n (%)	
Normal	21 (13.3%)
Overweight	87 (55.1%)
Obesity class I	36 (22.8%)
Obesity class II	12 (7.6%)
Obesity class III	2 (1.3%)
Smoking habits, n (%)	
Non-smoker	119 (75.3%)
Smoker	11 (7.0%)
Former smoker	28 (17.7%)
Diabetes mellitus, n (%)	36 (22.8%)
Dyslipidemia, n (%)	127 (80.4%)
Target organ damage (TOD) n (%)	46 (29.1%)
Type of TOD, n (%)	
Stroke	7 (4.4%)
Nephropathy	27 (17.1%)
Structural heart disease	20 (12.7%)
Atherosclerotic disease	9 (5.7%)
Retinopathy	2 (1.3%)
Reason for HBPM request, n (%)	
Follow-up	107 (67.7%)
Diagnostic	49 (31.0%)
Prior TOD	2 (1.3%)
Mean SBP, mmHg, mean ±​​​​​​​ SD	132.7 ± 15.7
Mean DBP, mmHg, mean ±​​​​​​​ SD	79.3 ± 8.5
HBPM results	
Follow-up group, n (%)	
Controlled HTN	39 (24.7%)
Uncontrolled HTN	66 (41.8%)
Low BP	2 (1.3%)
Diagnostic group, n (%)	
Hypertension	17 (10.8%)
White coat hypertension	32 (20.3%)
Previous TOD group, n (%)	
Normal BP	2 (1.3%)
Masked hypertension	0 (0.0%)
Pharmacological follow-up, n (%)	
Did not start drug therapy	38 (24.1%)
Started drug therapy	14 (8.9%)
Maintained drug therapy	53 (33.5%)
Changed drug therapy	53 (33.5%)
Dose increased	29 (54.7%)
Dose decreased	3 (5.7%)
Drug class changed	21 (39.6%)

Regarding the rationale for performing HBPM, 107 (67.7%) patients underwent the procedure for follow-up, 49 (31.0%) for diagnostic purposes, and two (1.3%) due to previous TOD for masked hypertension screening. Overall, the mean SBP was 132.7 ± 15.7 mmHg, and the mean DBP was 79.3 ± 8.5 mmHg. In detail, 39 (24.7%) had controlled hypertension, 66 (41.8%) had uncontrolled hypertension, two (1.3%) had low BP, 17 (10.8%) were newly diagnosed with hypertension, 32 (20.3%) had white coat hypertension, and two (1.3%) had normal BP and previous TOD. Regarding pharmacological treatment and considering the overall results, 14 (8.9%) patients initiated antihypertensive therapy, 38 (24.1%) did not start treatment, 53 (33.5%) continued their ongoing antihypertensive therapy, and 53 (33.5%) underwent changes in their treatment. Table 2 presents the distribution of comorbidities among the respective subgroups.

**Table 2:** Distribution of DM, dyslipidemia, and TOD by subgroups (HTN, white coat HTN, previously controlled HTN, previously uncontrolled HTN) DM: diabetes mellitus; TOD: target organ damage; HTN: hypertension

Group		DM, n (%)		Dyslipidemia, n (%)		TOD, n (%)	
		No	Yes	No	Yes	No	Yes
Diagnostic	HTN	17 (100.0%)	0 (0.0%)	5 (29.4%)	12 (70.6%)	15 (88.2%)	2 (11.8%)
	White coat HTN	28 (87.5%)	4 (12.5%)	10 (31.3%)	22 (68.8%)	31 (96.9%)	1 (3.1%)
Follow-up	Previously controlled HTN	32 (82.1%)	7 (17.9%)	6 (15.4%)	33 (84.6%)	30 (76.9%)	9 (23.1%)
	Previously uncontrolled HTN	43 (65.2%)	23 (34.8%)	9 (13.6%)	57 (86.4%)	35 (53.0%)	31 (47.0%)

Distribution of SBP and DBP

Table 3 presents a comparison between the distribution of SBP and DBP across the different diagnostic and follow-up groups. In the diagnostic group, mean SBP and DBP were significantly higher in patients with AH compared to those with white coat hypertension (SBP: 141.5 ± 12.9 mmHg vs. 122.3 ± 8.2 mmHg, p<0.001; DBP: 89.9 ± 5.2 mmHg vs. 76.8 ± 4.8 mmHg, p<0.001). In the follow-up group, patients with previously uncontrolled hypertension had a significantly higher mean SBP compared to those with previously controlled hypertension (142.9 ± 14.7 mmHg vs. 121.8 ± 7.7 mmHg, p<0.001); no statistically significant difference was found for DBP (82.1 ± 8.2 mmHg vs. 73.3 ± 5.7 mmHg, p=0.09).

**Table 3:** Comparison of mean SBP and DBP between diagnostic and follow-up groups SBP: systolic blood pressure; DBP: diastolic blood pressure; SD: standard deviation; AH: arterial hypertension

Groups		SBP, mean ± SD	P-value	DBP, mean ± SD	P-value
Diagnosis	AH	141.5 ± 12.9	<0.001	89.9 ± 5.2	<0.001
	White coat hypertension	122.3 ± 8.2		76.8 ± 4.8	
Follow-up	Previously controlled AH	121.8 ± 7.7	<0.001	73.3 ± 5.7	0.09
	Previously uncontrolled AH	142.9 ± 14.7		82.1 ± 8.2	

Therapeutic regimen of patients with prior hypertension before HBPM

Table 4 presents a comparison between the therapeutic regimen (monotherapy versus combination therapy with two or more drugs) of patients with previously controlled and uncontrolled hypertension, before performing HBPM. Among the subgroup of patients with previously controlled hypertension, 21 (53.8%) were on combination therapy and 18 (46.2%) were on monotherapy. In the subset of patients with previously uncontrolled hypertension, 43 patients (66.2%) were taking more than one antihypertensive drug, and 22 (33.8%) patients were on monotherapy.

**Table 4:** Comparison of the therapeutic regimen between patients with previously controlled and uncontrolled hypertension, prior to HBPM HBPM: home blood pressure monitoring; HTN: hypertension

Group (follow-up)	Monotherapy	Combination therapy	Total
Previously controlled HTN, n (%)	18 (46.2%)	21 (53.8%)	39
Previously uncontrolled HTN, n (%)	22 (33.8%)	43 (66.2%)	65
Total	40	64	104

Questionnaire assessment

Of the 82 patients who responded to the questionnaire, 49 (59.8%) belonged to the follow-up group and 33 (40.2%) to the diagnostic group. Table 5 presents the descriptive analysis of the responses to the applied questionnaire. Fifty-five (67.1%) patients stated that they considered hypertension a lifelong disease, and 82 (100%) stated that they believed it was possible to control this disease with diet and/or medication. Before performing HBPM, 47 (94.0%) patients reported taking their medication correctly at home, as prescribed by the physician. All patients (n=3) who reported non-adherence to the treatment plan stated that they began doing so correctly after performing HBPM.

**Table 5:** Questionnaire answers HBPM: home blood pressure monitoring; BP: blood pressure

Questions	N	Answers	N (%)
Is arterial hypertension a lifelong disease?	82	No	15 (18.3%)
		Yes	55 (67.1%)
		I don’t know	12 (14.6%)
Is it possible to control blood pressure with diet and/or medication?	82	No	0 (0.0%)
		Yes	82 (100.0%)
		I don’t know	0 (0.0%)
Before performing HBPM, did you take your medication correctly, as prescribed by your doctor?	50	No	3 (6.0%)
		Yes	47 (94.0%)
		I don’t know	0 (0.0%)
After performing HBPM, did you start taking your medication correctly, as prescribed by your doctor?	3	No	0 (0.0%)
		Yes	3 (100.0%)
		I don’t know	0 (0.0%)
Before HBPM, how often did you forget to take your hypertension medication?	49	Never	36 (73.5%)
		Once a month	10 (20.4%)
		Once a week	3 (6.1%)
		More than once a week	0 (0.0%)
After HBPM, how often do you forget your medication?	49	Never	43 (87.8%)
		Once a month	5 (10.2%)
		Once a week	1 (2.0%)
		More than once a week	0 (0.0%)
Did you have a blood pressure monitor at home?	82	No	30 (36.6%)
		Yes	52 (63.4%)
		I don’t know	0 (0.0%)
If yes, how often did you measure your BP?	52	Never	22 (42.3%)
		Once a month	21 (40 .4%)
		Once a week	6 (11.5%)
		More than once a week	3 (5.8%)
And how often do you measure your BP now?	52	Never	6 (11.5%)
		Once a month	23 (44.2%)
		Once a week	14 (26.9%)
		More than once a week	9 (17.3%)
Did you buy a blood pressure monitor for home use?	30	No	11 (36.7%)
		Yes	19 (63.3%)
		I don’t know	0 (0.0%)
If yes, how often do you measure your BP?	19	Never	3 (15.8%)
		Once a month	6 (31.6%)
		Once a week	8 (42.1%)
		More than once a week	2 (10.5%)
After performing HBPM, do you consider that you reduced the amount of salt in your diet?	82	No	32 (39.0%)
		Yes	47 (57.3%)
		I don’t know	3 (3.7%)
Before performing HBPM, did you practice any regular physical exercise (at least 30 minutes per day, 5 days a week)?	82	No	45 (54.9%)
		Yes	37 (45.1%)
		I don’t know	0 (0.0%)
After performing HBPM, did you start any regular physical exercise (at least 30 minutes per day, 5 days a week)?	82	No	34 (41.5%)
		Yes	48 (58.5%)
		I don’t know	0 (0.0%)
Did you consider performing this exam useful for your health?	82	No	2 (2.4%)
		Yes	78 (95.1%)
		I don’t know	2 (2.4%)

Regarding the frequency of missing antihypertensives pills, before HBPM, 36 (73.5%) patients reported never forgetting to take their medication, 10 (20.4%) reported forgetting them once a month, and three (6.1%) once a week. After HBPM, 43 (87.8%) patients reported never forgetting to take them, five (10.2%) reported forgetting them once a month, and one (2.0%) once a week. In no situation were forgetfulness episodes reported more than once a week. In 41 (83.7%) patients, the number of forgetfulness episodes remained the same; in eight (16.3%), the number decreased, and no patient reported an increase. The difference in forgetfulness frequency reported before and after HBPM was statistically significant (p=0.007).

Ownership of a sphygmomanometer at home was reported by 52 (63.4%) patients. Of those who already had one, 22 (42.3%) stated they had never measured BP before HBPM, 21 (40.4%) measured it once a month, 6 (11.5%) once weekly, and three (5.8%) more than once. After HBPM, six (11.5%) patients reported never measuring their BP, 23 (44.2%) reported monthly measurements, 14 (26.9%) reported weekly measurements, and nine (17.3%) reported more than once a week. In 21 (40.4%) patients, the measurement frequency remained the same; in 27 (51.9%), the frequency increased; and in four (7.7%), it decreased. The difference in measurement frequency before and after HBPM was statistically significant (p<0.001). After performing HBPM, 19 (63.3%) patients who did not previously own a sphygmomanometer stated they had purchased one.

Regarding salt intake, 34 (57.3%) patients reported reducing the amount of salt in their diet after performing HBPM. Before HBPM, 37 (45.1%) patients reported engaging in some form of physical exercise. This number increased to 48 (58.5%), post-HBPM. In 65 (79.3%) patients, physical exercise habits remained the same; in 14 (17.1%), they increased; and in three (3.7%), they decreased. The difference in reported physical exercise before and after HBPM was statistically significant (p=0.008).

## Discussion

In our sample, there was a slight predominance of the male participants (50.6%), and the mean age was 61.5 ± 12.8 years. As observed in other studies [13], there was a high prevalence of associated risk factors, namely overweight/obesity (86.7%), dyslipidemia (80.4%), DM (22.8%), association of dyslipidemia and diabetes (20.9%), and current or past smoking habits (24.7%). TOD was present in 46 patients (29.1%), with the highest proportion observed in the group with uncontrolled hypertension (47.0%). These findings are consistent with the literature, which reports a higher prevalence of TOD among individuals with poorly controlled AH [14]. Office BP values are frequently higher than HBPM values, leading to potential misdiagnosis of hypertension in 15-30% of cases. This phenomenon is known as white coat hypertension [5,15]. In our sample, this percentage was even higher: 65.3% of patients with elevated office BP had HBPM values suggestive of white coat hypertension. This highlights the importance of an early application of this tool to avoid unnecessary pharmacological interventions, reduce healthcare costs, and prevent iatrogenesis [16,17].

Beyond its diagnostic utility, HBPM proved to be a valuable method for monitoring BP in patients with a prior diagnosis of hypertension. In our study, 66 out of 107 (61.7%) patients already under AH follow-up were found to have uncontrolled BP. In these cases, therapeutic adjustments were made, which not only helped overcome therapeutic inertia but also enabled more precise treatment adjustments. Among patients under treatment, 53 (50.0%) had their therapy modified. Of these, 54.7% had a dosage increase and 39.6% experienced changes in pharmacological class. Although ABPM is the gold standard method for diagnosing AH, HBPM is a feasible alternative when ABPM is unavailable. In many outpatient healthcare providers, ABPM is not yet routinely accessible [18]. Several studies demonstrate a strong correlation between ABPM and HBPM, with sensitivity and specificity ranging from 60 to 90% in the diagnosis of AH, white coat hypertension, masked hypertension, and therapeutic follow-up of hypertensive patients [19].

Furthermore, HBPM has the advantage of being more accessible and accepted by patients. It is low-cost, easy to apply, and potentially more reproducible than office BP and ABPM [5,16]. Some studies have shown that patients who have received prior training are more likely to acquire and regularly use a home BP monitor [20]. In our sample, 75.5% of hypertensive patients and 45.5% of non-hypertensive patients already owned a BP monitor at the time HBPM was requested. These results indicate that the population included in our study was predisposed to perform HBPM, even though the monitor was not provided. However, the proportion of validated monitors already owned by patients remains unknown. Among patients who did not previously own a monitor, 63.3% purchased one. Most of them started using it regularly, showing good receptivity and acceptance of the method. In these cases, the benefit of the intervention extended beyond the time it was implemented.

Additionally, through self-monitoring and increased awareness of elevated BP values, HBPM promoted patient engagement [5,16], therapeutic adherence, and adoption of healthier lifestyle behaviors [5]. The adoption of these measures resulted from greater awareness, control, and patient empowerment, as perceived by 95.1% of those surveyed who felt the tool had benefited their health. Despite growing recognition that office BP alone is insufficient for diagnosing AH [21,22], a study involving physicians from 77 countries revealed that 87.0% still relied primarily on office BP values when deciding to prescribe antihypertensive therapy [23]. Several reasons contribute to this: the belief that many patients cannot perform HBPM correctly, especially those with low health literacy [24-26]; the use of non-validated devices [24,27]; and the possibility of anticipatory anxiety compromising the obtained values [22,24,27].

Poor adherence to good practices in HBPM leads to high rates of invalid measurements, which can compromise their interpretation [28]. To reduce the number of invalid HBPM in our study, instructions were given both verbally and in writing. A market analysis shows that fewer than 10% of BP monitors meet validation protocols [11]. To mitigate this potential error, we provided validated and calibrated monitors, which we consider a strength of our project.

This study presents several limitations. It involved a convenience sample from a single FHU, which may limit the generalization of the results. The information collected from medical records may be incomplete. Measurements were carried out by the patients themselves, so it is possible that some values were omitted, selectively chosen, or repeated to achieve the “expected” average. Moreover, the home measurement technique was not verified, and the same cuff size was used for all patients, which may have influenced the results, particularly in obese individuals. The form used for HBPM recording did not include a section for clinical notes, which would have allowed us to register symptoms suggestive of hypotension and information on therapy suspension during the monitoring period. The questionnaire was applied retrospectively, thus leading to a memory bias. Finally, the sample of patients who answered the questionnaire was relatively small, potentially reducing the statistical power of some of the results.

As opportunities for improvement, we recognize the importance of analyzing the full case series of the internal monitoring plan; use validated questionnaires and apply them before and after the intervention; include a section for observations and symptom reporting on the HBPM recording sheet; assess patient and physician satisfaction; and to verify whether there are repeated HBPM in the same patient and proceed with comparative analysis. Additionally, the implementation of supervised medication intake strategies, such as video consultations via the National Health Service app, may be beneficial for patients with poor BP control. Furthermore, our management approach may be strengthened by performing ABPM after HBPM in selected patients.

## Conclusions

This study reinforces the relevance of HBPM as a practical and effective tool in the management of hypertension in primary care. In addition to its diagnostic value, particularly in identifying white coat hypertension and cases of uncontrolled BP, HBPM has shown potential to promote patient empowerment, improve therapeutic adherence, and support the adoption of healthier lifestyle behaviors. By involving patients directly in their care, HBPM contributes to a more individualized and proactive approach to hypertension management, potentially reducing overtreatment and iatrogenic risk. Our findings support the integration of HBPM as a standard practice in primary care settings, particularly in contexts where ambulatory BP monitoring is not routinely available. Further prospective and multicenter research is needed to evaluate the long-term clinical impact, cost-effectiveness, and patient experience associated with HBPM, and to strengthen the evidence base for its integration as standard practice in primary care settings.
